# Minimum number and types of allergens for a skin prick test panel in Thai children with allergic respiratory diseases

**DOI:** 10.1186/s13223-022-00718-7

**Published:** 2022-08-24

**Authors:** Prapasri Kulalert, Orapan Poachanukoon, Sira Nanthapisal, Paskorn Sritipsukho, Karnsinee Thanborisutkul, Pasistha Termworasin, Rungrawee Kornsawai, Patcharaporn Punyashthira

**Affiliations:** 1 Department of Clinical Epidemiology, Faculty of Medicine, Thammasat University, Pathum Thani, 12120 Thailand; 2 Division of Allergy and Immunology, Department of Pediatrics, Faculty of Medicine, Thammasat University, Pathum Thani, Thailand; 3 Center of Excellence in Applied Epidemiology, Thammasat University, Pathum Thani, Thailand; 4 Center of Excellence for Allergy, Asthma and Pulmonary Disease, Thammasat University, Pathum Thani, Thailand

**Keywords:** Asthma, Allergic rhinitis, Allergens, Skin tests

## Abstract

**Background:**

Patterns of aeroallergen sensitization vary by countries. Testing with the minimum number of allergens is important to identify sensitized patients for a cost-effective approach. We aimed to assess the minimal skin prick test (SPT) panel to identify sensitized children with allergic respiratory diseases.

**Methods:**

The SPT results from January 2020 to December 2021 in children aged 2–18 years with symptoms of asthma or allergic rhinitis or both were retrospectively reviewed. All children received 11 allergen extracts (*Dermatophagoides pteronyssinus* [Der p], *Dermatophagoides farinae* [Der f], American cockroach, German cockroach, cat, dog, Bermuda grass, careless weed, Timothy, *Acacia*, and molds). The conditional approach was used to determine the allergen selection for the SPT panel.

**Results:**

A total of 688 children were enrolled (mean age = 8.14 ± 3.91 years). The sensitization results were Der p (57.85%), Der f (55.09%), German cockroach (18.02%), American cockroach (17.01%), cat (11.77%), *Acacia* (3.49%), Bermuda grass (3.34%), molds (3.05%), Timothy (2.33%), dog (1.89%), and careless weed (1.60%). Der p, Der f, and German cockroach were required to detect at least 95% of sensitized children. If the SPT panel added *Acacia*, cat, American cockroach, Bermuda grass, and careless weed, sensitization was detected in 99–100% of cases.

**Conclusions:**

Indoor allergens (Der p, cockroach, and cat) were common causes of sensitization in Thai children with allergic respiratory diseases. Eight allergens were sufficient for sensitization identification in Thai children with asthma or allergic rhinitis or both in clinical practice.

## Introduction

Asthma and allergic rhinitis are common allergic disease in children [1, 2]. The etiology of allergic diseases is complex due to a combination of genetics and interacting environmental factors [3, 4]. Aeroallergen exposure is closely linked to sensitization, which is a significant risk factor for the development, persistence, and severity of allergic respiratory disease in children [5–7]. Reducing environmental trigger exposure can decrease symptoms. Identification of allergen sensitization is essential for individualized allergen avoidance education and prescription of personalized treatment with specific allergen immunotherapy [8, 9].

The skin prick test (SPT) is the efficient in vivo test to demonstrate IgE-mediated sensitization in a patient with clinical history suggestive of asthma or allergic rhinitis or both. The SPT also demonstrates diagnostic accuracy in sensitized subjects [10, 11].

Differences were observed between countries with regard to the number and types of allergens for a SPT panel. Only eight to 10 allergens out of 18 allergens were sufficient to identify more than 95% of sensitized patients in a large multicenter study in 14 European countries (e.g., Austria, Belgium, Denmark, England, etc.) [12]. Determination of the minimum number and types of allergens for testing is important to identify most of the sensitized patients with a cost-effective approach [12, 13].

To the best our knowledge, a study to evaluate the optimal SPT panel to identify most of the sensitized children with symptoms that suggest asthma or allergic rhinitis or both is lacking in Thailand country. Therefore, the objective of this study was to determine the minimal number and type of SPT allergens required to identify sensitized children with symptoms of asthma or allergic rhinitis or both.

## Methods

### Study setting

This was a retrospective medical chart review of children who had the SPT from January 2020 to December 2021 at the Pediatric Allergy Clinic of Thammasat University Hospital, Pathum Thani Province, Thailand. Participants were identified by the SPT code T.111102 from our hospital database. They were required to have all of the following characteristics: (1) aged 2–18 years when they received the SPT; (2) diagnosed with allergic rhinitis or asthma or both; and (3) underwent a SPT for allergic respiratory diseases that was modified from a previous study at our hospital [14]. Children aged < 2 years were excluded due to the difficulty of making an accurate diagnosis of allergic rhinitis or asthma or both. Patients were excluded if their skin test result had no valid interpretation. A valid SPT result also required a positive histamine reaction (≥ 3 mm in diameter) and a negative control reaction (< 3 mm in diameter) [10, 15].

### Data collection

Patient age, gender, and diagnosis were recorded at the time of the SPT. The SPT results were collected. Our respiratory panel in current practice included 11 allergen extracts: *Dermatophagoides farina*e (10,000 AU/mL, AllerVACtest), *Dermatophagoides pteronyssinus* (10,000 AU/mL, AllerVACtest), dog epithelium (1:20 w/v, AllerVACtest), cat hair (10,000 BAU/mL, AllerVACtest), careless weed (1:30 w/v, AllerVACtest), mixed molds (1:10 w/v, ALK-Abelló)*, Periplaneta americana* (American cockroach, 1:20 w/v, ALK-Abelló), *Blattella germanica* (German cockroach, 1:20 w/v, ALK-Abelló), Bermuda grass (10,000 BAU/mL, ALK-Abelló), Timothy (10,000 BAU/mL, ALK-Abelló), and *Acacia* (1:20 w/v, ALK-Abelló). Histamine 10 mg/mL and glycerinated phenol-saline were used as positive and negative controls, respectively.

Our routine SPT panel originated from a previous study by Sritipsukho in our allergy clinic. Sritipsukho used 15 aeroallergen extracts that included *Dermatophagoides pteronyssinus*, *Dermatophagoides farina*e, American cockroach, German cockroach, cat dander, dog dander, Kapok, Bermuda grass, Johnson grass, *Acacia*, careless weed, *Alternaria*, *Penicillium*, *Aspergillus*, and *Cladosporium* [14]. However, extracts for individual molds, Kapok, and Johnson grass were not available at the time of this current study. Nevertheless, mixed molds composed of *Alternaria*, *Cladosporium, Drechslera sorokiniana, Aspergillus niger, and Penicillium*, and Timothy extracts were available. Therefore, a panel of 11 extracts is used in our current clinical practice.

The SPTs were performed at the Pediatric Allergy Clinic by well-trained technicians. All participants were instructed to cease antihistamine medication for seven days prior to the test. Wheal diameters were read 15–20 min after the test. Wheal size was measured by the longest and orthogonal diameters reported in millimeters (mm). Skin test positive was defined as a wheal diameter ≥ 3 mm larger than the negative control. Children with at least one positive SPT to an aeroallergen were considered to be sensitized.

### Statistical analysis

The statistical analysis used STATA version 14.0. Demographic characteristics were reported as percentage for categorical variables and mean ± standard deviation for continuous data. The prevalence of sensitization for each allergen was reported as percentage.

### Selection of the allergen panel

The step-by-step conditional approach was used to determine the allergen from the one that gave the highest increased in prevalence of sensitization to the one that gives the lowest [12, 16]. Initially, we identified the most prevalent allergen in all sensitized children. We then determined the next allergen that gives the highest increased prevalence of sensitization in the group of subjects not sensitized to the previous allergen. This procedure was repeated until none of the resulting allergens induced a change in prevalence. The optimal number and types of allergens were defined when a coverage of at least 99% for the presence of SPT sensitization was achieved. We performed the conditional technique for all children in the study. In addition, the same procedure was repeated with patients allocated by age group: 2 to < 6 years; 6 to < 12 years; and 12–18 years of age.

## Results

### Demographic and clinical characteristics

A total of 708 children with asthma or allergic rhinitis or both who underwent the SPT for aeroallergens were reviewed. Twenty (2.82%) were excluded due to histamine reaction that was < 3 mm in diameter. Finally, 688 children were enrolled in the analysis. The study included 413 (60.03%) males and 275 (39.97%) females with an overall mean age of 8.14 ± 3.91 years. Allergic rhinitis was diagnosed in 667 (96.95%) children and 209 (30.38%) children were diagnosed as asthma (Table 1).

**Table 1:** Characteristics of the study population

		Age group		
	Total	2 to < 6 years	6 to < 12 years	12–18 years
Characteristic	(*N* = 688)	(*N* = 241)	(*N* = 317)	(*N* = 130)
Age (years), mean ± SD	8.14 ± 3.91	4.18 ± 1.03	8.57 ± 1.68	14.43 ± 1.66
Gender				
Male	413 (60.03)	142 (58.92)	197 (62.15)	74 (56.92)
Female	275 (39.97)	99 (41.08)	120 (37.85)	56 (43.08)
Disease phenotypes				
Allergic rhinitis	667 (96.95)	224 (92.95)	314 (99.05)	129 (99.23)
Asthma	209 (30.38)	98 (40.66)	76 (23.97)	35 (26.92)

Data are presented as n (%) unless otherwise indicated

*SD* standard deviation

### Sensitization results

Table 2 shows the overall prevalence of sensitization to allergens in the study patients and the distribution of sensitization according to age group. Overall, the prevalence of SPT-positive to at least one allergen was 458 (66.57%). The sensitization rate to *Dermatophagoides pteronyssinus* (57.85%), was the highest, followed by *Dermatophagoides farinae* (55.09%), German cockroach (18.02%), American cockroach (17.01%), cat (11.77%), *Acacia* (3.49%), Bermuda grass (3.34%), molds (3.05%), Timothy (2.33%), dog (1.89%), and careless weed (1.60%).

**Table 2:** Prevalence of skin test sensitization to aeroallergens for all children and distribution according age group

	Total	2 to < 6 years	6 to < 12 years	12–18 years
	(*N* = 688)	(*N* = 241)	(*N* = 317)	(*N* = 130)
Sensitization rate	458 (66.57)	134 (55.60)	220 (69.40)	104 (80.00)
Der p	398 (57.85)	114 (47.30)	194 (61.20)	90 (69.23)
Der f	379 (55.09)	107 (44.40)	186 (58.68)	86 (66.15)
German cockroach	124 (18.02)	30 (12.45)	52 (16.40)	42 (32.31)
American cockroach	117 (17.01)	25 (10.37)	49 (15.46)	43 (33.08)
Cat	81(11.77)	18 (7.47)	35 (11.04)	28 (21.54)
*Acacia*	24 (3.49)	7 (2.90)	11 (3.47)	6 (4.62)
Bermuda grass	23 (3.34)	3 (1.24)	14 (4.42)	6 (4.62)
Molds	21 (3.05)	5 (2.07)	9 (2.84)	7 (5.38)
Timothy	16 (2.33)	2 (0.83)	9 (2.84)	5 (3.85)
Dog	13 (1.89)	4 (1.66)	7 (2.21)	2 (1.54)
Careless weed	11 (1.60)	2 (0.83)	5 (1.58)	4 (3.08)

Data are presented as n (%)

*Der p Dermatophagoides pteronyssinus*, *Der f Dermatophagoides farinae*

The prevalence of SPT positive to at least one allergen increased with age: 55.6% in the 2 to < 6-year age group, 69.40% in the 6 to < 12-year age group, and 80.00% in the 12–18-year age group. Moreover, the prevalence of the SPT reactivity to each allergen tended to increase with age. The 3 most common allergens of all age groups were house dust mite (*Dermatophagoides pteronyssinus* and *Dermatophagoides farinae*), cockroach (German cockroach and American cockroach) and cat.

Table 3 shows the minimum test panel to reach a detection rate of 95–100% of sensitized children in all ages and in different age groups. For all ages, we found that only three allergens (*Dermatophagoides pteronyssinus*, *Dermatophagoides farinae*, and German cockroach) were adequate to identify at least 95% of all sensitized children under 18 years of age. When the five allergens of *Acacia*, cat, American cockroach, Bermuda grass, and careless weed were added to the previous three allergens, sensitization was detected in 99–100% of the cases.

**Table 3:** The test panel for all children according to age group

	*n*	%	Cumulative %
Total (*N* = 458)			
Der p	398	86.90	86.90
German cockroach	23	5.02	91.92
Der f	15	3.27	95.19
* Acacia*	8	1.75	96.94
Cat	6	1.31	98.25
American cockroach	3	0.65	98.90
Bermuda grass	2	0.44	99.34
Careless weed	2	0.44	99.78
Dog	1	0.22	100
Children 2 to < 6 years (*n* = 134)			
Der p	114	85.07	85.07
German cockroach	8	5.97	91.04
Der f	5	3.73	94.77
* Acacia*	4	2.99	97.76
Careless weed	2	1.49	99.25
Bermuda grass	1	0.75	100
Children 6 to < 12 years (*n* = 220)			
Der p	194	88.18	88.18
Der f	10	4.55	92.73
German cockroach	6	2.73	95.46
Bermuda grass	5	2.27	97.73
Cat	2	0.91	98.64
American cockroach	2	0.91	99.55
Dog	1	0.45	100
Children 12–18 years (*n* = 104)			
Der p	90	86.54	86.54
German cockroach	6	5.77	92.31
Cat	4	3.85	96.16
Der f	2	1.92	98.08
American cockroach	1	0.96	99.04
* Acacia*	1	0.96	100

*Der p Dermatophagoides pteronyssinus*, *Der f Dermatophagoides farinae*

The number and types of allergens were slightly different between the age groups. The SPT panel with five allergen extracts for preschool children (*Dermatophagoides pteronyssinus*, *Dermatophagoides farinae*, German cockroach, *Acacia*, and careless weed), six allergens for school aged children (*Dermatophagoides pteronyssinus*, *Dermatophagoides farinae*, German cockroach, Bermuda grass, cat, and American cockroach), and five allergens for adolescents (*Dermatophagoides pteronyssinus*, *Dermatophagoides farinae*, American cockroach, German cockroach, and cat) were needed to detect 99% of the sensitized children. However, the selected eight allergens (*Dermatophagoides pteronyssinus*, *Dermatophagoides farinae*, German cockroach, *Acacia*, cat, American cockroach, Bermuda grass, and careless weed) in the SPT panel were adequate to detect to 99–100% for all age group.

## Discussion

Identification of allergen sensitization in each country is necessary to provide comprehensive knowledge of locally prevalent allergen sensitization, which should be helpful in personalized education in allergen avoidance and management of an allergic disease. In addition, determining the minimum number and types of allergens needed to test is a cost-effective approach. However, the prevalence of allergen sensitization in Thai children has not been studied.

In the present study, we identified eight allergens (*Dermatophagoides pteronyssinus*, *Dermatophagoides farinae*, German cockroach, American cockroach, cat, *Acacia*, Bermuda grass, and careless weed) that were sufficient to identify 99–100% of sensitized children under 18 years of age with symptoms suggestive of allergic rhinitis or asthma or both. Our results were consistent with previous studies that found only eight to nine allergens were sufficient to identify allergen sensitization in children [16–18]. However, the types of allergens were different in different countries and geographies [19].

Sahiner et al. reported that the most common allergen sources in Turkey were grass, followed by house dust mite, cat, weeds, and *Alternaria* species. Testing with nine allergen extracts (*Festuca pratensis*, *Dermatophagoides pteronyssinus*, *Phleum pratense*, *Alternaria alternata*, cat, *Lolium perenne*, *Dermatophagoides farinae*, *Cynodon dactylon*, and cockroach) identified approximately 95% of allergen sensitization in Turkish children under 18 years old with suspected respiratory allergy [17]. These results differed from our results. We showed that the three most common causes of allergen sensitization in Thai patients were indoor allergens, which were house dust mite followed by cockroach and cat. Grass and pollen had lower sensitizations than indoor allergens. Only three allergen extracts, which included *Dermatophagoides pteronyssinus*, *Dermatophagoides farinae*, and German cockroach, were adequate to identify at least 95% of all sensitized children under 18 years of age. If we increased the number of extracts to eight to include *Acacia*, cat, American cockroach, Bermuda grass, and careless weed, sensitization was detected in 99–100% of the cases.

Wang et al., who performed SPTs in China, included children and adults with allergic rhinitis. They found that the predominant aeroallergen was dust mite with a sensitization rate of over 90% in the whole group, while other allergens, such as pollen, mold, and cockroach, affected less than 10%. Their results were similar to the prevalence results of our study. We reported house dust mite as the most common sensitization (approximately 85%), while other allergens, such as pollen, mold, and cockroach, affected less than 10% in sensitized children. However, the selection of allergens in the panel by Wang et al. was different. In a panel of three allergens, they used *Dermatophagoides farinae*, *Dermatophagoides pteronyssinus*, and *Platanus*, which provided a positive sensitization rate > 95%. In a panel of eight allergens, they used *Dermatophagoides pteronyssinus*, *Dermatophagoides farinae*, *Platanus*, *Artemisia*, *Cryptomeria*, *Blattella germanica*, *Humulus*, and *Alternaria*, which was sufficient to identify over 99% of sensitized patients suffering from allergic rhinitis symptoms in Central China [18].

A recent study from Jordan reported only eight allergen extracts were necessary to identify 95% of the sensitized patients. The eight allergen extracts used in children with allergic rhinitis or asthma or both were olive pollen, *Dermatophagoides pteronyssinus*, *Salsola kali*, cereals, wall pellitory, *Dermatophagoides farinae*, cypress, and *Alternaria* [16].

In this current study, the number and types of allergens in the minimum test panel differed slightly among age groups, which was consistent with other studies [17, 20]. However, the optimized panel of eight allergens for Thai children was able to identify 99–100% of sensitized children.

The house dust mite was the most common sensitization, followed by cockroach and cat. The other allergens including pollen, weed, dog dander, and mold affected less than 5% in these children. Indoor allergens are common in Thai children, which is consistent with previous studies in Thailand [14, 21, 22].

The eight allergens selected for this study were optimized for Thai subjects in clinical practice. The panel of three allergens (*Dermatophagoides pteronyssinus*, *Dermatophagoides farinae*, and German cockroach) can be used for the initial screening in areas with limited budgets. The results of this study suggest using the additional five extracts (*Acacia*, cat, American cockroach, Bermuda grass, and careless weed) for Thai children who tested negative in the initial screening, which was only about 5% of children in the initial screening in this study. However, the patterns of allergen sensitization are unique in each country. Therefore, physicians in each country or geographical region need to establish the optimal number and types of allergens for screening.

The strength of this study is that this is the first study to determine the minimal number and type of allergen extracts for a SPT panel to detect allergen sensitization in Thai children with allergic respiratory disease. This study demonstrated that only eight allergens were appropriate for detection in sensitized children. It reduces the number of test allergens used in current practice, which can also reduce both costs and physician workload, not to mention the discomfort of the needle prick in children.

The limitations in this study need to be stated. First, we focused only on sensitization and did not explore the clinical relevance of sensitization and symptoms. However, the retrospective chart review limited our ability to gather data on the clinical relevance. A future prospective study would be helpful. Second, our study aimed to identify the minimal number of allergens in the SPT panel to achieve the highest positive rate; therefore, some allergens were not included (e.g. dog). If patients report clinical relevance to an allergy, they should be tested. Finally, a cost effectiveness analysis was not performed, which requires further study.

In summary, indoor allergens (dust mites, cockroaches, and cats) were common causes of sensitization. Our study showed that the eight extracts of *Dermatophagoides pteronyssinus*, *Dermatophagoides farinae*, German cockroach, *Acacia*, cat, American cockroach, Bermuda grass, and careless weed were the optimal allergens for the SPT in Thai children with allergic rhinitis or asthma.

## Data Availability

The dataset analyzed in the current study is available from the corresponding author on reasonable request.

## Abbreviations

SPT: Skin prick test
Der p: *Dermatophagoides pteronyssinus*
Der f: *Dermatophagoides farinae*
